# Editorial: Prion-Like transmission of pathogenic proteins in neurodegenerative diseases: structural and molecular bases

**DOI:** 10.3389/fmolb.2023.1294465

**Published:** 2023-09-21

**Authors:** Victor Banerjee, Fei Wang, Daniela Sarnataro, Claudio Soto

**Affiliations:** ^1^ Department of Neurology, Mitchell Center for Alzheimer’s Disease and Related Brain Disorders, Houston McGovern Medical School, University of Texas Health Science Center, Houston, TX, United States; ^2^ Department of Molecular Medicine and Medical Biotechnologies, University of Naples Federico II, Naples, Italy

**Keywords:** prion-like propagation, PMCA, neurodegenerative diseases, sialylation, prion strain

Understanding the propagation of pathogenic proteins in various neurodegenerative diseases has become a pivotal focus of research. Prion-like mechanisms are at the heart of these investigations, spanning diseases such as Alzheimer’s disease (AD), Parkinson’s disease (PD), dementia with Lewy bodies (DLB), multiple system atrophy (MSA), and more. This Frontiers in Molecular Biosciences Research Topic, entitled “Prion-like transmission of pathogenic proteins in neurodegenerative diseases: structural and molecular bases,” brings together four distinct contributions from experts who delve into different facets of neurodegenerative disorders. These studies collectively explore the complexities and interconnections between post-translational modifications, the risks of transmission through contaminated blood, innovative prion propagation techniques, and the structural understanding of amyloid filaments in neurodegenerative diseases. Together, they provide comprehensive insights into the multifaceted nature of neurodegeneration and the evolving strategies to address these challenging conditions.

The studies featured in this Research Topic provide profound insights into the realm of neurodegenerative diseases and the underlying prion-like mechanisms that drive their progression. Makarava et al. studied the effect of sialylation in the pathogenesis of prions diseases. Sialylation, the post-translational modification involving the addition of sialic acid to proteins, emerges as a pivotal factor influencing the rate of prion replication and its consequences for the host organism. Manipulating the sialylation state of PrP^Sc^, the abnormal prion protein, by modifying the sialylation of its precursor, PrP^C^, holds promise in the battle against prion diseases. Makarava et al. used a mouse model with a global knockout of ST6Gal1, one of the two sialyltransferases in mammals responsible for catalyzing the glycan sialylation. Notably, this knockout resulted in a significant reduction of sialylation in the brain tissue. However, it is worth noting that the sialylation of PrP^Sc^ remained largely unaltered, and the observed change in the disease incubation time in ST6Gal1 knockout mice did not reach statistical significance. The study raises thought-provoking questions regarding the roles of specific sialidase genes, the potential involvement of alternative sialyltransferases like ST6Gal2, and the impact of age-related changes in sialylation on sporadic Creutzfeldt-Jakob disease (CJD) and regional vulnerability to prion infections.

Additionally, concerns regarding the transmission of CJD between individuals through prion-contaminated blood products are addressed. A study by Jaffre et al. involving cynomolgus macaques exposed to such contaminated blood reveals an unexpected myelopathic syndrome predominantly affecting the spinal cord. This research identifies a unique biochemical signature supporting the prion origin of this syndrome, challenging conventional understanding. Notably, it involves abnormal PrP forms that are less protease-resistant than regular PrP^Sc^, posing complexities in their detection and emphasizing the need for reevaluation of a possible prion involvement in various diseases, especially those affecting the spinal cord. This study suggest that prion diseases may in some cases manifest with dramatically different features as the classical disorders included in the group of transmissible spongiform encephalopathies.

Traditionally, researchers have relied on animal models expressing the prion protein, for the study of prion diseases. While informative, these studies come with significant costs, long-term animal care requirements, and ethical concerns. To address these challenges, the development of a technology known as protein misfolding cyclic amplification assay (PMCA) has been instrumental. PMCA enables the rapid and efficient propagation of prions *in vitro*, amplifying them within days. Importantly, PMCA maintains infectivity and the unique properties of prion strains, offering a viable alternative to animal experiments for prion production. Concha-Marambio et al. reported the development and optimization of a large-scale PMCA (LS-PMCA) to produce massive amounts of infectious prions on a large scale. LS-PMCA presents promising applications in prion structural studies by enabling the investigation of natural prions from various species as well as possibly expanding its use to other misfolded proteins linked to neurodegenerative diseases, such as AD, PD, DLB, and MSA. This not only advances our understanding of prion diseases but also reduces the dependence on animal experiments, addressing both scientific and ethical concerns.

Lastly, the work by Gram et al. delves into the importance of studying brain-derived amyloid filaments to better understand common neurodegenerative diseases. Typically, researchers use the detergent sarkosyl to solubilize brain fractions and isolate sarkosyl-insoluble filamentous species for structural and functional studies. Sarkosyl, considered a mild detergent that effectively extracts fibrillar proteins from brain tissue, has been widely used in research. However, this study highlights that some populations of αSyn aggregates, which exhibit different sensitivity to sarkosyl, may have been lost during extraction. Additionally, the study points out that the process of freezing and thawing brain tissue, a common practice in brain banks, can significantly affect the stability of αSyn filaments, particularly in the cases of PD. Similarly, sonication, another common step in tissue processing, can destabilize PD filaments. This destabilization leads to the conversion of filaments into soluble seeding-competent aggregates, without modifying strain-specific characteristics. In conclusion, this research underscores the importance of considering the differential stability of αSyn aggregate strains during sarkosyl extraction. It suggests that variations in stability, influenced by factors like freezing and sonication, can impact the outcome of studies examining these aggregates. Researchers may need to explore alternative methods to better capture the diversity of αSyn aggregates in neurodegenerative diseases.

In summary, these studies collectively shed light on the intricate connections between prion diseases, prion-like propagation and the challenges encountered in the study of neurodegenerative disorders. They advocate for innovative approaches to study these diseases and being aware of the current limitations on the research studies. This will be important to advance our comprehension of these diseases and potentially unveil new therapeutic avenues.

